# Improved outcomes of pediatric patients with vascular malformations treated at a multidisciplinary vascular anomaly clinic

**DOI:** 10.1016/j.jdin.2025.07.002

**Published:** 2025-07-17

**Authors:** Sofía Valdés-Loperena, Andrea González-Moreno, Itzel Guadalupe Elizalde-Jiménez, Carola Durán-McKinster, María Teresa García-Romero

**Affiliations:** Instituto Nacional de Pediatría, México City, Mexico

**Keywords:** child, epidemiology, patient care team, treatment outcome, vascular malformations

*To the Editor:* Diagnosis and management of vascular malformations (VaMs) is challenging, even for experienced physicians.^1^ We aimed to study patient outcomes after being treated at referral sites, at a tertiary institution, and at a multidisciplinary vascular anomaly clinic (VAC).

This was a retrospective study of consecutive patients treated at the VAC from January 1, 2012 to October 25, 2023. We categorized patients into simple, combined, of major vessels, associated syndromes (ASs), and provisionally unclassified VaMs.^2^ We analyzed outcomes according to a composite investigator global assessment (IGA) from 0% to 100% including radiological, clinical, and/or patient-reported response. We compared outcomes before referral, at our tertiary institution, and at the VAC using Wilcoxon signed-rank test.

We included 527 patients (277, 52.5% were female) and most (372, 70.5%) had simple malformations (Table I). The median age at diagnosis was 1 (range 0-180 months) months. Common signs and symptoms were increased volume (513, 97.3%) and color change (341, 64.7%). Most patients had multiple locations affected (183, 34.7%) and predominant locations were trunk (178, 33.7%) and face/head (164, 31.1%).

**Table I:** Demographic and clinical characteristics of 527 included patients with vascular malformations (VaMs)

	*n* (%)
Median age at initial signs and symptoms (range)	1 (0-180) mo
Median age at diagnosis (range)	77.5 (0-219) mo
Type of VaM∗	
Simple	372 (70.5%)
Lymphatic†	201 (54%)
Venous‡	111 (29.8%)
Arteriovenous malformations/fistulae§	49 (13.1%)
Capillary	11 (2.9%)
Combined	124 (23.5%)
Lymphatic-venous malformation	94 (75.8%)
Capillary-venous malformation	18 (14.5%)
Capillary-lymphatic-venous malformation	9 (7.2%)
Capillary-lymphatic malformation	2 (1.6%)
Lymphatic-arteriovenous malformation	1 (0.8%)
Associated syndromes	25 (4.7%)
Klippel-Trenaunay syndrome	16 (64%)
Proteus syndrome	2 (8%)
Servelle-Martorell syndrome	2 (8%)
Bannayan-Riley-Ruvalcaba syndrome	1 (4%)
CLAPO syndrome‖	1 (4%)
CLOVES syndrome¶	1 (4%)
Maffucci syndrome	1 (4%)
Provisionally unclassified	6 (1.1%)
Fibro adipose vascular anomaly	6 (100%)
Location	
≥1 location	183 (34.7%)
Trunk	178 (33.7%)
Head/face	164 (31.1%)
Lower extremities	149 (28.2%)
Neck	110 (20.8%)
Upper extremities	104 (19.7%)
Signs and symptoms	
Volume increase	513 (97.3%)
Color change	341 (64.7%)
Pain	233 (44.2%)
Functional disability	185 (35.1%)
Bleeding or oozing	53 (10%)

*VaM*, Vascular malformation.

∗ We did not include any patients with the ISSVA 2018 classification category of VaM of major named vessels.

† Common lymphatic malformations (LMs) (198, 53.2%), generalized lymphatic anomaly, Kaposiform lymphangiomatosis, and LM in Gorham-Stout disease (1, 0.2% patient each).

‡ Common venous malformations (106, 28.4%), blue rubber bleb nevus syndrome (5, 1.3%) patients.

§ Arteriovenous malformations (48, 12.9%) and arteriovenous fistula in 1 (0.2%).

‖ Capillary malformations of the lower lip, lymphatic malformations of face/neck, asymmetry of face and limbs, and partial or generalized overgrowth.

¶ Congenital lipomatous overgrowth, vascular malformations, epidermal nevi, and spinal/skeletal anomalies.

Before referral to and at the tertiary institution, only 103 (19.5%) and 112 (21.2%) patients were treated, respectively (Table II). Once at the VAC, 419 (79.5%) patients received treatment; sclerotherapy and combined therapy the most frequent.

**Table II:** Treatments and outcomes of 527 patients with all types of VaM at 3 sites

	Before referral	*P* value∗	Tertiary institution	*P* value†	VAC	*P* value‡
Treatments used						
Sclerotherapy	17 (16.5%)	-	41 (36.6%)	-	236 (56.3%)	-
Surgery	22 (21.3%)	-	27 (24.1%)	-	14 (3.3%)	-
Medical treatment	32 (31%)	-	13 (11.6%)	-	47 (11.2%)	-
Embolization	1 (0.9%)	-	3 (2.6%)	-	10 (2.3%)	-
Combined	31 (30%)§	-	28 (25%)‖	-	112 (26.7%)¶	-
Average number of treatments per patients						
All VaM	0.44 ± 0.77	**<.001**	0.39 ± 0.68	**<.001**	1.14 ± 0.82	**<.001**
Simple	1.12 ± 1.86	**<.001**	0.54 ± 1.55	**<.001**	2.06 ± 2.53	**<.001**
Combined	0.98 ± 1.64	**.02**	0.67 ± 1.65	**<.001**	2.60 ± 3.11	**<.001**
AS	0.88 ± 1.83	-	1.08 ± 2.62	.18	1.64 ± 1.84	**.01**
PU	5.83 ± 0.75	.15	0.16 ± 0.40	.19	1.33 ± 1.96	.65
Average improvement by IGA						
All VaM	20.4% ± 30.84	.195	47.6% ± 31.6	**<.001**	73.2% ± 27.0	**<.001**
Simple	23.9 ± 34.1	.191	47.3 ± 30.1	**.002**	76.8 ± 24.8	**.003**
Combined	15.7 ± 22.6	.58	48 ± 35.3	**.007**	67.9 ± 28.1	**.003**
AS	6 ± 13.4%	.65	41.6 ± 38.1	.31	55 ± 36	.10
PU		-		-	85 ± 21.2	-
Recurrences						
All VaM	1.37 ± 1.07	.18	1.66 ± 1.91	.41	1.49 ± 2.06	.10
Simple	1.37 ± 1.20	.317	1.96 ± 2.26	.41	1.75 ± 2.54	.31
Combined	1.42 ± 0.87	.31	1.33 ± 1.41	1.00	1.03 ± 0.19	.31
AS	1.2 ± 0.44	-	1 ± 0.00	-	1 ± 0.00	-

Bolded values are significant *P* values.

*AS*, Associated syndrome; *IGA*, investigator global assessment; *PU*, provisionally unclassified; *VAC*, vascular anomalies clinic; *VaM*, vascular malformation.

∗ Pairwise comparison between before referral and tertiary institution values.

† Pairwise comparison between tertiary institution values and VAC.

‡ Pairwise comparison between before referral and VAC.

§ Surgery + sclerotherapy in 18 (58%) patients, surgery + medical treatment and sclerotherapy + medical treatment in 5 (16.1%) each one, and surgery + sclerotherapy + medical treatment in 3 (9.6%).

‖ Surgery + sclerotherapy in 16 (57.1%) patients, sclerotherapy + embolization in 5 (17.8%), surgery + medical treatment in 4 (14.2%), surgery + embolization in 2 (7.1%), and sclerotherapy + medical treatment in 1 (3.5%).

¶ Sclerotherapy + medical treatment in 49 (43.7%) patients, surgery + sclerotherapy in 21 (18.7%), sclerotherapy + embolization in 12 (10.7%), surgery + Sclerotherapy + medical treatment in 9 (8%), surgery + embolization in 7 (6.2%), surgery + medical treatment in 5 (4.4%), sclerotherapy + embolization + medical treatment in 3 (2.6%), medical treatment + embolization in 3 (2.6%), surgery + embolization + medical treatment in 2 (1.7%), and surgery + sclerotherapy + embolization in 1 (0.8%).

Patients received more treatments at the VAC (1.14 ± 0.82 compared to 0.44 ± 0.77 before referral and 0.39 ± 0.68 at the tertiary institution, *P* < .001). Subanalysis showed these findings were significant for simple, combined, and ASs.

Mean improvement after treatment at the VAC was 73.2% ± 27.0%, greater than before referral and at the tertiary institution (20.4% ± 30.84% and 47.6% ± 0.68, respectively, *P* < .001). By categories, these results remained for patients with simple and combined VaM.

In recent years, interdisciplinary programs have been established to provide diagnosis and treatment to patients with VaM. In this retrospective study of 527 children with VaM, we found patients with simple, combined, and ASs received a higher number of treatments at the VAC, and combination therapies tended to be more diverse.

Surgical approaches, historically preferred to treat VaMs, have been replaced by modalities like sclerotherapy and medical targeted treatments (such as mammalian target of rapamycin inhibitors), especially for combined VaMs and ASs.^3^, ^4^, ^5^ Types and treatment combinations elected at the 3 time points in this study reflect these variations: 21.3% to 24.1% of patients received surgery before referral to the VAC compared to 14% afterward.

We found greater improvement in composite IGA in patients with simple and combined VaMs treated at the VAC, which could be explained by their higher prevalence and greater medical expertise to treat them, but also due to more and diverse treatments.

Recurrence rates were similar across sites, although data from before referral to the VAC were limited. Other limitations include the retrospective methodology and the current lack of standardized outcome measures for VaMs, reason for which we used a self-derived composite IGA.

Our results highlight that multidisciplinary VACs may lead to a higher number and more diverse treatment choices as well as enhanced patient outcomes, suggesting patients with VaMs should be referred to these centers.

## Conflicts of interest

None disclosed.
